# Rectal neuroendocrine tumor during anorectal surgery: three case reports and a review of the literature

**DOI:** 10.3389/fonc.2025.1468266

**Published:** 2025-03-17

**Authors:** Ruoxi Dong, Jingen Lu, Hao Zhou, Qingjun Dong, Chen Wang

**Affiliations:** Department of Anorectal Surgery, LongHua Hospital, Shanghai University of Traditional Chinese Medicine, Shanghai, China

**Keywords:** rectal neuroendocrine tumors, surgery, diagnosis, treatment, case report

## Abstract

**Background:**

Rectal neuroendocrine tumors (NET) are thought to originate from the diffuse neuroendocrine system. The lack of apparent signs of illness and the patient’s non-specific presentations often cause a delay in diagnosis, until in their final stages of cancer. Thus, rectal NETs pose a significant challenge to most physicians.

**Case presentation:**

This article presents three cases of rectal NETs discovered during anorectal surgery. Owing to their atypical symptoms, they were initially diagnosed as mixed hemorrhoids or perianal fistulas. However, the patients were diagnosed with rectal NETs and thus underwent endoscopic dissection or transanal endoscopic microsurgery. Histological analysis revealed three rectal NETs, one G1 and two G2. All patients were followed-up for more than 6 months, with excellent outcomes without recurrence.

**Conclusions:**

The etiology, pathogenesis, therapeutic methods, prevention, and prognosis of rectal NETs remain challenging. Given the variable understanding of the most appropriate operative approaches for rectal NETs, our objective was to broaden the perspective of this infrequent disease by delivering distinctive individual experiences and emphasizing the therapeutic significance of delicate surgery.

## Introduction

Rectal neuroendocrine tumors (NET), which are thought to originate from the diffuse neuroendocrine system, are a fusion of neurologic and endocrine features (1). Most of rectal-NETs are asymptomatic, a possibly cause for the increase of incidence rate may be a consequence of more elaborate and numerous screening colonoscopies with widespread use of endoscopy for detecting colorectal cancers (2). Despite diagnostic modalities, the diagnosis of rectal NETs is not straightforward; it may present with symptoms such as bleeding or changes in bowel habits, or without any at all (3). Several techniques are available for resecting rectal NETs, including the endoscopic, trans-anal and surgical therapies (4). Endoscopically resecting consists of endoscopic mucosal resection, endoscopic submucosal dissection (ESD), and endoscopic full-thickness resection (5). But the lack of apparent signs of illness and the patient’s non-specific presentations often result in delayed diagnosis, until in their advanced disease stages (6). Furthermore, the macroscopic appearance of rectal NETs resembles that of hyperplastic or adenomatous polyps, making the differential diagnosis from other polypoid lesions challenging (7). Thus, rectal NETs pose a significant challenge to most physicians. Herein, we present three cases of rectal NETs. Our objective was to further emphasize the diagnostic value of endoscopy and digital rectal examination for detecting rectal NETs as well as to enhance patient cognition of this disease, thereby providing insights and high reference values for both imaging radiologists and clinicians.

## Case report

### The first case

**Figure 1:**
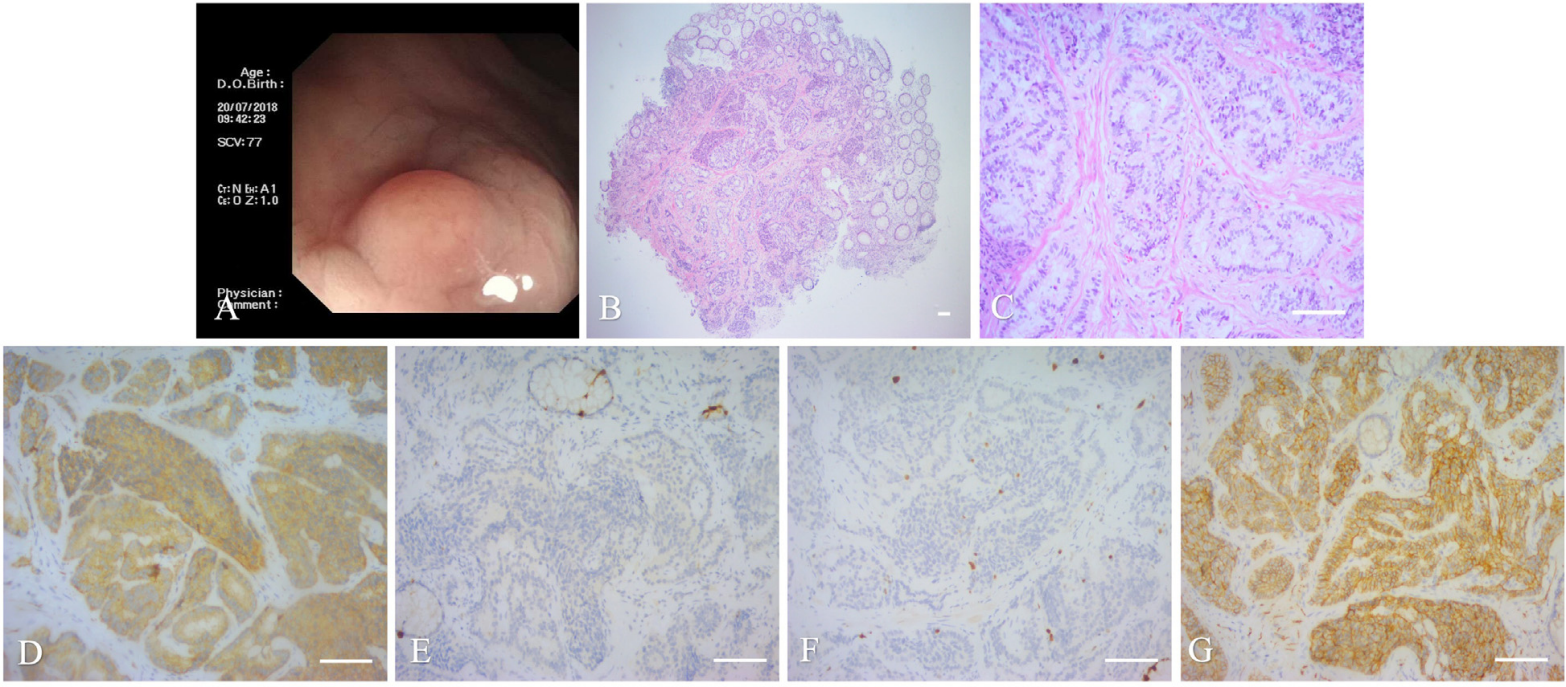
Imaging and histopathology of the biopsy specimen of the first case. **(A)**: Colonoscopy showing a mass approximately 0.5×0.5 cm in size. **(B, C)**: Histopathology of the mass (magnification, ×40 and ×200, scale bar of 100 μm). **(D–G)**: IHC of the mass, scale bar of 100 μm). **(D)** Cancer cells positive for Syn (Syn; ×200). **(E)** Cancer cells negative for CgA (CgA; ×200). **(F)** Cancer cells positive for Ki-67 (Ki-67, ×200). **(G)** Cancer cells positive for CD56(CD56; ×200).

A 46-year-old male presented with lumps and pain in the perianal and -rectal regions. The lump ruptured with spontaneous drainage for 1 year. Physical and laboratory examinations showed no obvious abnormality. An indentation, located 1 cm above the dentate line at the 12 o’clock direction in the bladder lithotomy position, was associated with slight tenderness as well as with a soft, subcutaneous induration extending to the rectum. Before the surgery, a colonoscopy was done, showing a 5mm×5mm longitudinal, protruding mass in the lower posterior rectal wall. Colonoscopic examination had also shown the lesion region, shape, and scope. Thus, surgeons have chosen proper operative schemes, wherein endoscopic resection was an effective treatment for mass resection without impairing the functional anatomy and biochemical of the anorectal physiological condition. After obtaining patient consent, ESD was performed, wherein the tumor was excised en bloc using a disposable, high-frequency knife. To date, doctors remain unaware of any adverse events associated with this dissection. Histologic hematoxylin and eosin (HE) staining confirmed a rectal NET, having a tumor stage of G1 (WHO 2019), revealing atypical epithelial cells. Immunohistochemistry (IHC) also demonstrated the diagnosis of a rectal NET, as the pathologic tissue section was positive for Synaptophysin(Syn), negative for chromogranin A(CgA), and with approximately 2% Ki-67 expression (**Figure 1**). Long-term follow-up over 5 years showed no recurrence, with an excellent outcome.

### The second case

A 29-year-old female initially presented with occasional, reducible prolapses during defecation with a 3 year-long progression, later progressing to recurrent, irreducible prolapse. The patient was diagnosed with mixed hemorrhoids. The patient had no family history of colorectal cancer, a past medical history of hashimoto thyroiditis for 15 years, and a medication history of thyroxine intake.

Laboratory tests, including routine blood work, liver and kidney function tests, coagulation function, and electrocardiography, were all unremarkable. Serum tumor marker findings, such as carcinoembryonic antigen(CEA), squamous cell carcinoma, carbohydrate antigen 72-4(CA72-4),19-9(CA19-9), 50(CA50), 242(CA242), 211, alpha-fetoprotein(AFP), and neuron-specific enolase(NSE) were all negative. Pre-operative colonoscopy findings were also unremarkable. Physical examination revealed mixed stage III hemorrhoids and rectal polyps. The patient underwent mixed hemorrhoidectomy and rectal polypectomy. Engorged hemorrhoidal cushions were observed at the 12-, 6-, and 7-to 8 o’clock positions in the bladder lithotomy position. During hemorrhoidectomy, a gray, 5 mm×5 mm×3 mm, hyperplastic lesion located at the dentate line at the 6 o’clock position. The surgeon then incised the rectal mucous membrane and carefully separated the lesion from the rectal muscle stratum. Finally, the intact hyperplastic lesion was completely resected using a high-frequency electrotome. A shuttle-like incision was designed to be incorporated to avoid creating narrow skin bridges and to protect the rectal muscle, in order to maintain the integrity of the rectal mucosa. Then, the muscular layer of the rectum was closed by the surgeons via a horizontal mattress suture.

**Figure 2:**
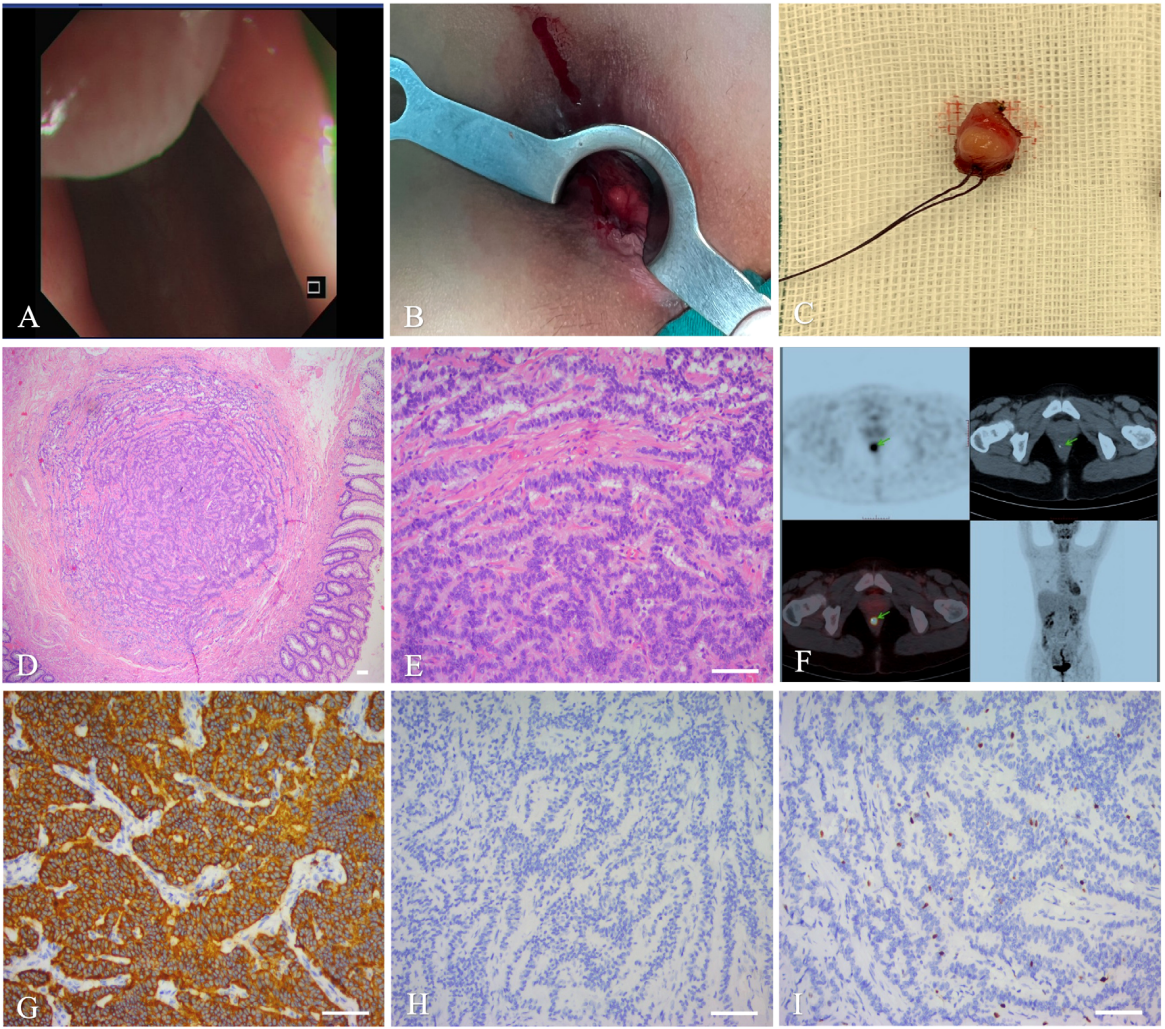
Imaging and histopathology of the biopsy specimen of the second case. **(A)**: Colonoscopy. **(B)**: Intra-operative photographs. **(C)**: Operative specimen. **(D, E)**: Histopathology of the mass (magnifications,×40, ×200, scale bar of 100 μm). **(F)**: PET-CT. **(G–I)**: IHC showing Syn **(G)**, CgA **(H)**, and Ki-67 **(I)**, scale bar of 100 μm.

The final pathologic findings included a tumor measuring 3 mm× 3 mm × 3 mm in size as well as atypical epithelial cells in the rectal submucosa and rectal muscle, but with tumor-free margins. HE staining confirmed a rectal NET, having a tumor stage of G2. Several mitotic figures and necrotic images were observed in the pathologic sections. IHC indicated that the tumor cells were positive for neuroendocrine markers, including Syn, somatostafin receptor 2(SSTR2), and insulinoma-associated protein1(INSM1), and with an approximately 8% Ki67 expression, but were negative for CgA and CDx2 (**Figure 2**). After surgery, the patient underwent delayed positron emission tomography-computed tomography (PET-CT) examination, wherein the tumor was then staged as T1aN0M0, phase I, with no distant metastases. Therefore, surgery alone was sufficient enough to cure the cancer without the need for subsequent adjuvant therapies. Over the 6-month follow-up period post-surgery, the patient did not experience any anal dysfunction or evidence of tumor recurrence.

### The third case

A 74-year-old male was admitted with anal and rectal lumps for 1 month, worsening over 3 days without hematochezia. The patient was initially diagnosed with rectal polyps. Family history for colorectal cancer was negative. Recurrent episodes of anal fistulas were present in the three patients, all of whom had a prior history of anal fistula surgery. However, this patient had no history of surgery nor trauma.

Laboratory tests revealed a hemoglobin concentration of 128 g/L,WBC count of 10.70*10^9^/L, platelet count of 282 × 10^9^/L, and normal C-reactive protein levels. Hepatic, renal, and blood coagulation functions were also unremarkable. Standard serum tumor markers, AFP, CA19-9, CA72-4, CA50, and CA242 were also unremarkable. However, CEA (6.1 ng/mL) and NSE were elevated (25.30 ng/mL).

After admission to our hospital, a series of surveys were performed, including colonoscopy, endoscopic ultrasonography, transrectal ultrasound, and perianal magnetic resonance imaging (MRI). Transrectal ultrasound showed a 26 × 11-mm, solid, hypoechoic lesion in the lower rectal segment. Endoscopic ultrasonography revealed a solid nodule at the right wall of the rectal mucosa with associated rectal muscularis propria thickening, for which endoscopic ultrasound-guided fine needle aspiration was performed as necessary. Iso-intense, perianal MRI showed a 95-mm oval tumor, located at the upside canal mucosa and about 3.5 cm from the anus, and a 2.2×1.5-cm irregular tumor, located at the right rectal mucosal wall and about 7-8 cm from the anus. The canal lesion showed iso-intense signals on T1- and T2-WI, while the rectal lesion showed a hypointense signal on T1WI and a slightly hyperintense one on T2WI. The lesions showed significantly low or inhomogeneous enhancement after perianal MRI reinforcement.

Enhanced MRI of the lower abdomen revealed a malignant tumor in the right rectal wall with multiple lymph node metastases. The patient underwent colonoscopy and biopsy and was then diagnosed with a rectal NET. PET-CT scan was also performed before the surgery to understand the delayed uptake of ^18^F-fluoro-2-deoxy-D-glucose by the mass over-time. The goal of PET-CT was to further clarify the location and characteristics of the mass, as well as to detect long-distance metastasis appropriate tumor staging. Right lower rectal wall radioactivity and adjacent lymph node swelling increased, with multiple liver metastases.

**Figure 3:**
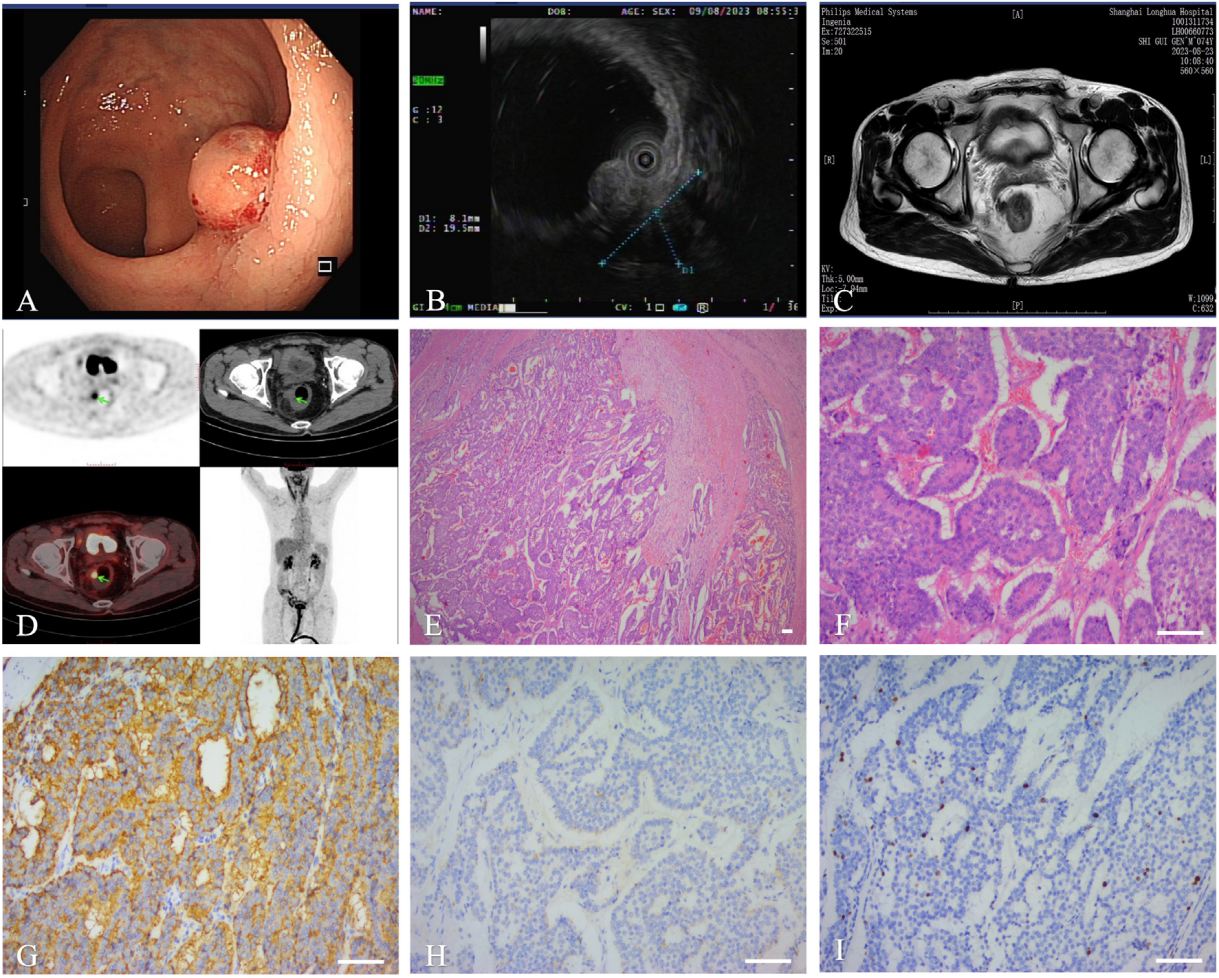
Imaging and histopathology of the biopsy specimen of the third case. **(A)**: Colonoscopy. **(B)**: Ultrasound colonoscope. **(C)**: MRI. **(D)**: PET-CT. **(E, F)**: Histopathology showed that differentiated(magnifications,×40, ×200, scale bar of 100 μm). **(G–I)**: IHC showing Syn **(G)**,CgA **(H)**, Ki-67 **(I)**, scale bar of 100 μm.

The patient underwent laparoscopic tumor resection with rectal lymph node dissection under general anesthesia. During surgery, the surgeons found that the mass compressed the adjacent bowel, with contracture of the sigmoid colon mesentery. To improve the patient’s quality of life and to prolong survival, the primary tumor was completely resected to prevent recurrence, wherein subsequent stenosis or an ileal stoma was then constructed. HE staining of the tumor on post-operative pathological examination revealed atypical epithelial cells. IHC staining showed a positive reaction for Syn, SSTR2, INSM1, and CgA, an approximately 10% Ki-67 expression, and a negative reaction for CDx2 (**Figure 3**). Based on these pathologic and imaging characteristics, a diagnostic staging of a pT4N1Mx rectal NET was established. Post-operatively, the patient underwent a 5-day course of antibiotic treatment. The patient recovered without complications and was discharged on post-operative day 14.

**Figure 4:**
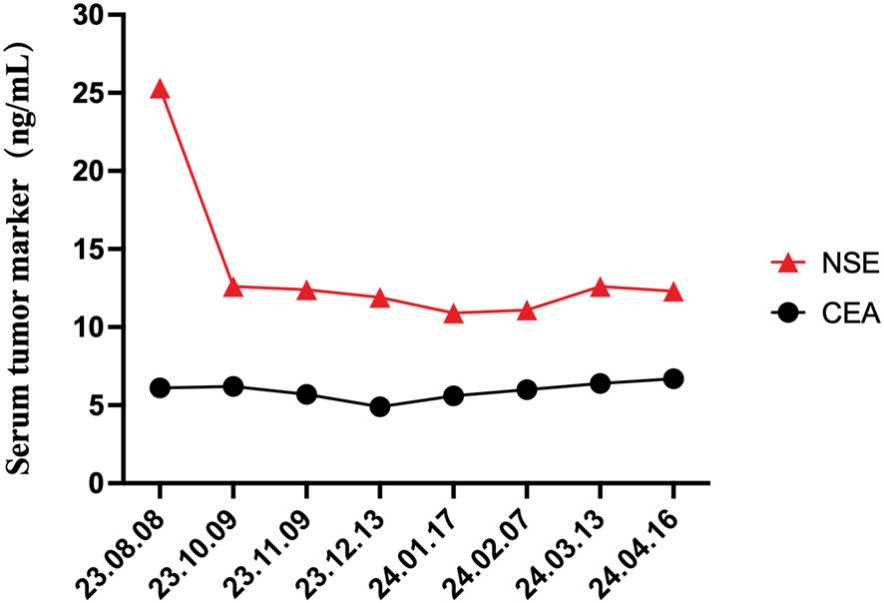
Changes in the serum tumor markers, NSE and CEA, during the treatment and throughout the 6-month follow-up periods.

Given this advanced stage with a high degree of malignancy and a non-functioning tumor behavior, the physicians recommended a treatment strategy using first-line targeted therapy, Surufatinib 300mg daily for days 1 to 28. Over the 6-month follow-up period, there were no significant side effects, clinical or radiological manifestations of local recurrence, nor distant metastases (**Figure 4**).

## Discussion

NETs, originating from sensory and secretory neuroendocrine cells, can arise in multiple organs and are the most common endocrine tumors of the gastrointestinal (GI) tract (8, 9). NETs were once called carcinoids, and carcinoid tumors were first described by Langhans in 1867 (10). However, almost half a century later in 1914, Gosset and Mason recognized that although the structure and histology of carcinoids were similar to those of cancer, their biological behavior was completely different. Carcinoids show slow progression of pathological changes and are essentially endocrine-related tumors (11).

NETs stems from the neural crest cells—a group of cells that can differentiate into various cell types, including the specialized neuroendocrine cells. The principal reason for NET incidence correlates strongly with the distribution density of neuroendocrine cells because these tumors originate from neuroendocrine cell compartments. Several transcribed genes are implicated in tumor development, metastasis and hormone secretion and can be useful in defining primary NETs and predicting metastasis occurrence. These genes include Ki-67 proliferation index, nucleosome assembly protein 1- like, CgA, INSM1 (12). Serum CgA has been the general biomarker for well-differentiated NETs for a long time. Its diagnostic accuracy is now under debate. However, it is useful in follow-up. A new immunohistochemical marker is the transcription factor INSM1, which is more specific for the differentiation of NETs of the pancreas and rectum (13).

The intestines, considered as the body’s largest endocrine organ, may arise nearly 60% of NETs. The region in the human body with the highest NET incidence rate is the GI tract (67.5%), followed by the bronchopulmonary system (25.3%); the remaining 10% occur in the liver, kidneys, and other endocrine glands. Researches show that within the GI tract, the small intestine (41.8%) is the most common location, followed by the rectum (27.4%) and stomach (8.7%) (14). NETs are usually considered rare. 1%–2% of all rectal tumors are neuroendocrine (15).However, over the past decades, their incidence has increased in most countries (16). According to the Surveillance, Epidemiology, and End Results (SEER) database in the US, showed a 10-fold increase of incidence in rectal NETs over the past 30 years in the United States (17), the same trend was also confirmed in the German registry (18), as well as in the Asian registers (19).

The US research data indicated that the greatest incidence of pathogenic sites for all gastro-entero-pancreatic NETs (GEP-NETs) are the rectum (29.2%) and small intestines (28.4%); in China, GEP-NETs were most frequently found in the pancreas (31.5%) and rectum (29.6%). The total number of rectal NET cases has also progressively improved by approximately 8%. Significant sex differences also exist in the incidence of rectal NETs, with rectal and pancreatic NETs occurring more commonly in men (20, 21).

The basic classification of NETs is based on the mitotic count and Ki-67 index, and is classified into three grades (G1, G2, and G3) that can be applied to almost any part of the GI tract: well-differentiated NETs with benign or uncertain behavior, well-differentiated neuroendocrine carcinomas with low-grade malignancy, and poorly differentiated neuroendocrine carcinomas. According to their degree of Ki-67 labeling index, malignant tumors can be categorized into three pathological grades: 1% to 3% as low risk; 3% to 20% as intermediate risk; more than 20% as high risk.

CgA, a member of the neuropeptide family, may assess the activities of the neuroendocrine system and is widely distributed in the neuroendocrine system. Several documentations indicated that CgA may be regarded as a tumor marker because its expression among patients could be increased to 70% to 90% (22). Among two present cases in this series, the specimens were all positive for CgA. Irregular mitosis are closely connected with the degree of cellular differentiation (23). Pathological and immunohistochemical analyses remain as the gold standard for diagnosis, whereas CgA and Syn are often selected as biological NET markers owing to their sensitivity and specificity (24).

Research has shown that rectal NETs are usually, but not exclusively, small and generally of low-to-intermediate grades (G1 or G2) (25). Given the substantial differences between common GI adenocarcinomas and NETs in terms of pathological physiology and prognosis, clinicians have instituted into account the individualized therapeutic methods that consider the biology of NET lesions (14). Currently, if there is no evidence of invasion beyond submucosa and presence of locoregional disease, endoscopic treatment for rectal NETs is indicated (26), usually complete tumor resection remains as the best curative option for patients with NETs (27, 28).

According to the 2016 European Neuroendocrine Tumor Society (ENETS) and 2020 National Comprehensive Cancer Network (NCCN) guidelines, treatment protocols for rectal NETs should vary according to their size, grade, and stage. For example, for G1 or G2 and stage T1 lesions with a diameter of 1–2 cm, the 2016 ENETS guidelines suggest minimally invasive surgery, whereas the NCCN guidelines recommend endoscopic resection. For those with a diameter of less than 1 cm, the ENETS guidelines recommend endoscopic resection as an effective treatment. R0 resection is an effective method for primary tumors and does not require follow-up for patients, which received support from the NCCN guidelines. Furthermore, for R0 lesions with a diameter of less than 1 cm, current guidelines are capable of creating treatment benefits for patients without further follow-up monitoring (29). Guidelines from the ENETS recommend local excision via a transanal or endoscopic approach for rectal NETs of less than 1 cm, and radical resection for those greater than 2 cm (25).

In these three cases, we found that the symptoms of rectal NETs were atypical, making misdiagnosis inevitable (**Table 1**). Therefore, there is greater demand for the physicians’ clinical skills. Ultrasonic contrast can also be used for further evaluating rectal NETs, if necessary. Based on our experiences, we recommend complete resection for all rectal-NETs. For rectal-NETs <1 cm without muscularis propria invasion, endoscopic resection may be a viable treatment option. For the rectal-NETs >2 cm, or with lymph node involvement, or grade 3 histology require radical surgical resection. Although our case report is based on three patients, compared with relevant guidelines, both our surgical methods and treatment procedurals of the of rectal NETs have some defects that need perfecting. Even the patient was diagnosed as anorectal disease, it is still necessary to perform colonoscopy preoperatively. Even when colonoscopy is performed preoperatively, digital rectal examination before surgery is indispensable and can provide more intuitive information regarding the NET location, size, shape, density, margins, and relationship with its surrounding tissues, which are important for operation schemes. We believe that there are different surgical methods in effective tumor control and could also improve the overall health of these patients. As reported in these cases, those different size of NETs require collaboration between different departments for better treatment. In our case report, endoscopic resection is enough for smaller rectal-NETs, bigger one should receive surgery therapy from anorectal surgery department. In addition, according to rectal NET grading and genotyping tests, molecular targeted therapy, chemotherapy, peptide receptor radionuclide therapy, and immune checkpoint inhibitor therapy may also enhance therapeutic potential. We also need more photo/video documentation to describe rectal NETs in more detail. Moreover, because the surgery number are few, we still have to accumulate more case of rectal NETs and the experience, to further observe its forward curative effect.

**Table 1:** Clinical characteristics of reported cases of rectal-NETs.

Variable	Patient 1	Patient 2	Patient 3
Sex	Male	Female	Male
Age(y)	46	29	74
Symptoms	Lumps and pain in the perianal and -rectal regions	Recurrent, irreducible prolapse during defecation	Anal and rectal lumps for 1 month, worsening over 3 days without hematochezia.
Laboratory tests	Normal	Normal	Hb:128g/L,WBC:10.70*10^9^/L, PLT:282×10^9^/L, CEA:6.1ng/mL,NSE:25.30ng/mL
Imaging tests	Colonoscopy showing a 5mm×5mm longitudinal, protruding mass in the lower posterior rectal wall	Direct vision shows a gray 5mm×5mm ×3mm, hyperplastic lesion located at the dentate line at the 6 o’clock position	PET-CT shows that right lower rectal wall radioactivity and adjacent lymph node swelling increased, with multiple liver metastases
Tumor stage	G1, T1aN0M0 phase I	G2, T1aN0M0 phase I	G2, pT4N1Mx
Treatment	Endoscopic submucosal dissection (ESD)	Transanal resection	Laparoscopic tumor resection with rectal lymph node dissection,with targeted therapy(Surufatinib)
Outcome	Survive with no recurrence	Survive with no anal dysfunction or evidence of tumor recurrence	Survive with no significant side effects, clinical or radiological manifestations of local recurrence, nor distant metastases.

From the lungs to the gastrointestinal tract and the pancreas, neuroendocrine tumors can emerge in almost any body part, each site offering its unique challenges and management paradigms (30). A clear understanding of the tumor’s organ of origin and its inherent characteristics is indispensable for determining the most suitable therapeutic approach. Moreover, the highlighted controversies, especially in the importance of accurate diagnosis and tailored management strategies that require further exploration and consensus-building. Ultimately, the goal is to provide personalized, effective care that enhances patients’ quality of life and survival outcomes.

## Conclusion

Rectal NETs are often accompanied by symptoms of bleeding or disruption of bowel habits. However, almost half of the patients produce very few noticeable symptoms. It is easily misdiagnosed as a benign anorectal disease; therefore, it is crucial to perform colonoscopy preoperatively. Endoscopic or transanal resection of rectal NETs has a good overall prognosis. Recent studies have suggested that a histological grade of G2 may be rated as an independent risk factor for R1 resection. Further follow-up management after rectal NET R0 resection is also necessary.

## Data Availability

The original contributions presented in the study are included in the article/supplementary material. Further inquiries can be directed to the corresponding authors.
